# The potential competency outcome of vigorous brain training game in the elderly: A systematic review

**DOI:** 10.1002/hsr2.1406

**Published:** 2023-07-26

**Authors:** Karunrat Tewthanom, Sittha Sukasi, Sarawut Lerspalungsanti, Foifon Srisawat

**Affiliations:** ^1^ Department of Pharmaceutical Care, Faculty of Pharmacy Silpakorn University Meaung Thailand; ^2^ National Metal and Materials Technology Center Khlong Nueng Thailand

**Keywords:** potential outcomes, systematic review, vigorous brain training game

## Abstract

**Background and Aims:**

The potential outcomes of vigorous brain training game in the elderly is questionable.

**Methods:**

A systematic review of studies reporting the outcomes of brain training game under the PRISMA guideline was conducted using the PubMed database (1997–2022). The selection criteria were clinical studies published in English language.

**Results:**

A total of 174 articles were identified by searching keywords. However, after screening the relation of the topic and methodology, 21 articles were included. The results of all studies showed positive outcomes after using the brain training game. Variation in the measurement tools were observed.

**Conclusion:**

The brain training game showed a positive effect on the brain function. However, the confirmation studies with large populations and standard measurement tools are required for more validated results.

## Key points

- The variations of measuring the effect of vigorous brain training game on brain function in elderly were observed.
- Most studies provided positive impact on potential outcomes.
- Standardized measurement methods and well‐designed have been considered.

## 1 INTRODUCTION

Information worldwide revealed that many countries, including Thailand, are entering the aging era. According to the Elderly Situation Report in 2017,^1^ Thailand has a population of Serpentine 60, 11 million people a year or more, that is 17% of the population (65.5 million people). Thailand is experiencing a very rapid increase in the number of elderly people. In less than 4 years, Thailand will become a completely aging society. As the proportion of people aged 60 and over rises by 20%, the aging population in Thailand is growing rapidly.^1^

As the “millionaire population wave” born during 1963–1983 is moving to become an elderly person, in the next 20 years, Thailand will have an elderly population of up to 20 million, and most importantly, the late elderly population (aged 80 and over) will increase dramatically from 1.5 million in 2017 to 3.5 million over the next 20 years. The potential of these seniors should be developed such that they will not become a burden on their children and to avoid wasting the budget for caring for the effects of various diseases that are often found in the elderly, such as dementia and cerebrovascular diseases^1^, ^2^

The vigorous brain training game is a brain training program that may be useful for the elderly brain function; for example, computer‐based game, the activities that aim to exercised brain. Several studies have provided evidence that the use of games will contribute to the development of various potential of the elderly.^3^, ^4^, ^5^ However, there are a few systematic reviews disclosing the overview picture of advantages and disadvantages of the vigorous game in developing the potential competency in the elderly. Therefore, this study aims to provide a systematic review the influence of vigorous brain training games on elderly brain function outcome.

## 2 METHODS

### 2.1 Systematic review process

#### 2.1.1 Keywords

The searching keywords of systematic search were brain training game, older, elderly, and competency.

#### 2.1.2 Prisma systematic review guide process

**Figure 1:**
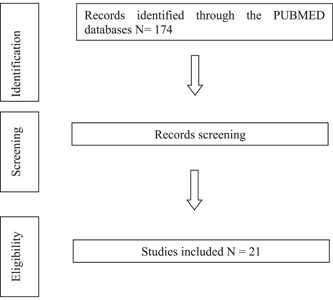
Preferred Reporting Items for Systematic Reviews and Meta‐Analyses process.

This systematic review process was aligned with the Preferred Reporting Items for Systematic Reviews and Meta‐Analyses (PRISMA) guidelines as presented in Figure 1 The selection criteria were clinical studies published in English. The population included the elderly. The outcome was measure of effectiveness of brain stimulation game by various tools. Unrelated articles or unclear measurement tools were excluded. After the articles were retrieved, their content was screened based on the relevant topic. All recruited studies were summarized.

## 3 RESULTS

### 3.1 Literature review results

#### 3.1.1 Identification

By using keywords as mentioned in the method section, 174 articles were identified.

#### 3.1.2 Screening

After the process of identification, the researchers read and evaluated the relation of the research questions, population, and methodology. The 153 articles were excluded because of unrelated topic.

#### 3.1.3 Eligibility

The 21 articles were recruited, the detail of each recruited studies illustrated in Table 1. All studies tended to have positive outcome in results of brain training program from various designs and measurements.

**Table 1:** Summary of studies about vigorous brain training game in the elderly.

Study	*N*	Method	Outcome
Lennart et al.^6^	21 elderly (65–90 years) 19 young adults (18–25 years)	In a controlled field study, the study used a 2 × 2 mixed factorial design (age group: young and old, game form: paper and Nintendo DS) to examine the effects of age and game form on usability, self‐evaluation, and gameplay experience. Efficiency as error rate, self‐assessment measures (arousal, enjoyment, domination), and gaming experience were all included when evaluating effectiveness (challenge, flow, competence, tension, positive and negative affect).	Using pen and paper instead of a Nintendo DS system is more effective and efficient for all players, regardless of age. For players of all ages, the game is more stimulating and creates a greater sense of flow when played digitally. Digital games that require players to solve logic problems may evoke pleasant emotions in older players but negative emotions in younger players. According to the author, aging Western civilizations may benefit from playing digital logic‐training games.
Nouchi et al.^7^	32 (completed = 28)	This double‐blinded randomized control trial compared the effects of Tetris and the brain training game Brain Age on seniors. The study's hypothesis was hidden from both participants and testers. The frontal assessment battery at bedside (FAB) and the mini mental status examination (MMSE) were utilized as measurement instruments.	The findings suggested that playing Brain Age for 4 weeks could help seniors' cognitive abilities (executive abilities and processing speed) improve.
Lee et al.^8^	31 (control = 16, intervention = 15)	The effectiveness of computer‐based cognitive training games for 8 weeks was explored in this pilot randomized control research. The difference between the system's pre‐ and posttraining total score on the repeatable battery for the assessment of neuropsychological status (RBANS) was used to gauge its effectiveness.	RBANS scores did not statistically differ between arms; an effect size of 0.6 SD was found. The median change in total scores between pre‐ and posttraining was similarly statistically significant, according to combined data from both arms (Median changed = 4.0; *p* < 0.05). The scores for immediate memory (*p* = 0.038), visuospatial/constructional (*p* = 0.014), attention (*p* = 0.039), and delayed memory (*p* = 0.001), all showed a substantial improvement.
Anguera et al.^9^	Experiment 1 = 90 Experiment 2 = 60 Experiment 3 = 18	Online and newspaper advertisements were used to find all of the participants. Experiment 1: Neurosignals were recorded while multitasking performance was assessed using a cost index. In the second experiment, it was determined whether older persons who trained by playing NeuroRacer could multitask effectively. In Experiment 3, older adults' midline frontal theta power and long‐range coherence were compared to those of a naive group of younger adults who had not received any training.	Increases in midline frontal theta power were found to be predictive of the training‐induced boost in sustained attention and the maintenance of multitasking improvement 6 months later. The results showed that this training had performance benefits that extended to untrained cognitive control abilities (i.e., improved working memory and sustained attention).
Choi et al.^10^	Intervention group *N* = 12, control group *N* = 12	Tweeny four patients who had suffered ischemic strokes were enrolled in this double‐blind, randomized experiment. The mobile upper extremity rehabilitation program employing a smartphone and tablet PC was used for 30 min in the intervention group in addition to traditional occupational therapy (OT) for 30 min (MoU‐Rehab). The controls (*n* = 12) only got traditional occupational therapy for 1 h each day. Ten treatment sessions spread across 5 days a week for 2 weeks made up the rehabilitation process. (Fugl−Meyer Assessments of the upper extremity (FMA‐UE, B‐stage for the arm and hand, manual muscle testing, modified Barthel index, EQ‐5D, and Beck Depression Inventory) were performed before and after treatment as well as after 1 month. A questionnaire was used to measure user satisfaction.	After using the MoU‐Rehab as opposed to traditional therapy, a larger improvement in the FMA‐UE, B‐stage, and MMT was observed. There was no discernible difference between the two groups in the magnitude of improvements on the MBI, EQ‐5D, and BDI. The experimental group's patients finished the 2 weeks of treatment without experiencing any negative side effects, and they were generally happy with MoU‐Rehab.
Eggenberger et al.^11^	42	Three 30‐min sessions each week were scheduled for the 8‐week intervention. On a treadmill, preferred, rapid walking pace was recorded, and prefrontal cortex (PFC) activity was measured. Shifting, inhibition, and working memory were evaluated as executive functions.	The findings demonstrated a substantial reduction in left and right hemispheric PFC oxygenation during the acceleration of walking for both Interventions (*p* = 0.05 or trend, *r* = 0.25–0.36), with DANCE showing a bigger reduction. After 30 s of walking, the left PFC was compared to BALANCE [*F* = (1,31)3.54, *p* = 0.035, *r* = 0.32]. Improvements in executive function were associated with these exercise training‐induced changes in PFC oxygenation (*p* = 0.05 or trend, *r* = 0.31–0.50).
Ballesteros et al.^12^	55 (control = 25, intervention = 30)	This study makes use of a repeated measures pretest, posttest, and 6‐month follow‐up experimental group with an active control group and a single‐blinded randomized controlled trial. Seventy‐five senior citizens in good cognitive health were divided into the experimental and active control groups at random. Sixteen 1‐h training sessions using cognitive nonaction video games from the commercial brain training program Lumosity were given to participants in the experimental group. The Sims and SimCity, a simulation strategy game, were used for the same amount of training sessions with the active control group. Behavior and electrophysiological tests were used to measure attentional networks. Two groups (experimental and active control) will be used in variance analyses at three different time points (pretest [baseline], posttest [postintervention], and follow‐up [after 6 months without contact]).	The main findings were: (1) neither the experimental group nor the active control group demonstrated greater gains in measures of selective attention or working memory than did the oddball task; (2) a marginal training effect was seen for the N‐back task but not for the Stroop task, while both groups made gains in the Corsi Blocks task. These findings suggest that training with nonaction games offers only marginal advantages for tasks that have not yet been trained. Since a comparable effect was noted for strategic video games, the effect is not unique to that type of training. In terms of expectations, involvement, or motivation, groups did not differ.
Yeo et al.^13^	Control = 121, intervention = 119	A 60–80‐year‐old, 0–0.5 clinical dementia rating (CDR) score, 24 or higher on the MMSE, and undiagnosed neuropsychiatric subject in a randomized controlled experiment. The waitlist‐control group or the Intervention group was chosen at random for each participant. The training program BRAINMEM includes games for working memory, delayed recall, and attention. The intervention regimen included 24 sessions spread out over 8 weeks and booster sessions every 3 months. The Repeatable Battery for the Assessment of Neuropsychological Status (RBANS) total score following the 24‐session training was the main result.	After the intervention, there were no discernible between‐subjects variations in general cognitive function. There was, however, a sex moderation effect (*p* = 0.014). Men in the intervention group outperformed those in the waitlist group in terms of performance. However, both waitlist participants who were female showed improvement from baseline, even though the between‐group difference in improvement was not statistically significant. Participants gave BRAINMEM favorable reviews and adhered to the intervention.
Al‐Thaqib et al.^3^	51	Fifty‐one individuals in good health were chosen to participate in a 3‐week experiment using a computerized cognitive training game called Lumosity to practice a variety of executive functions, such as attention, processing speed, and visual memory. The control group (*n* = 21) skipped the training. Before and 3 weeks after training for various cognitive processes, both groups took the CANTAB test (flexibility, memory, attention, speed, and problem solving). The levels of apolipoprotein E (APOE) and brain‐derived growth factor (BDNF) were examined in serum samples.	By the end of the training, the active group's Lumosity performance index had significantly improved compared to the control group (*p* = 0.001). Following the training, there was a statistically significant difference in the majority of the CANTAB measurements, including the MOT mean correct latency, attention‐switching task (AST), mean correct latency, AST switching cost, mean correct latency (congruent), mean correct latency (incongruent), and AST mean correct latency (Blocks 3 and 5 [nonswitching blocks]). However, no appreciable gains were seen in the control group. Pattern recognition memory (PRM) and APOE were found to be positively correlated, and those with greater APOE levels responded more quickly.
Perrot et al.^14^	Control = 11, KBT = 12, SMB = 12	Thirty‐six senior citizens were divided into three groups at random: Kawashima Brain Training (KBT), Super Mario Bros. (SMB), and the no‐training, no‐contact control group. All participants underwent a battery of cognitive tests, including ones for numerical comparison, spatial relationship, Corsi clock, digit symbol substitution, and matrix reasoning. Then, over the course of 2 months, participants in the game groups were told to play the videogame (KBT or SMB) for an hour, three times per week, for a total of 24 h of training. The three groups again took the cognitive tests following the 24 1‐h game sessions.	The matrix reasoning change score was considerably higher in both gaming groups compared to the control group, according to Tukey's post hoc tests and the analysis of variances on each of the cognitive measures. In comparison to the control and SMB groups, the KBT group's Stroop test change was noticeably higher. In comparison to the control group with KBT intermediate, the SMB group exhibited a considerably larger change in the DSST, Corsi block test, spatial relations test, and number comparison test.
Schättin et al.^15^	58	A parallel, double‐blind, randomized controlled experiment that lasted 26 weeks randomly assigned 58 participants to one of two groups (*N* = 29 for each group). Daily fish oil was given to the experimental group whereas daily olive oil was given to the control group. Both groups began an exercise game after 16 weeks. Measurements were taken before, throughout, and after the intervention. Transcranial magnetic stimulation recruitment curves and electroencephalography response‐locked potentials were the main results. Gait metrics and executive functioning were secondary results. Blood samples were collected to monitor fatty acids (FAs).	The study was completed by 43 people, with a mean age of 69.4 4.6 years (*N* experimental = 22, *N* control = 21). The findings revealed that no parameter had any discernible time–group interaction effects. Blood samples showed measurable effects of time and group interaction. For the experimental group, post‐hoc analyses revealed a significant rise in omega‐3 FAs. (*p* < 001) and a significant decrease in omega‐6 FAs (*p* < 001).
Jirayucharoensak et al.^16^	Control = 54, intervention = 65	Fifty‐four older women in good health and 65 women with mild cognitive impairment (MCI) were recruited. All participants received standard care (CAU); 58 patients received CAU + NFT (20 sessions of 30 min each, 2–3 sessions per week); 36 patients received CAU + exercise‐based training; and 25 patients received simply CAU. The Cambridge Neuropsychological Test Automated Battery was used to evaluate cognitive abilities both before and after therapy.	When compared to exergame training and no active treatment, neuro feedback training (NFT) dramatically enhanced spatial working memory (SWM), including strategy. Impaired SWM (including strategy), memory for pattern recognition, and delayed matching to samples were the hallmarks of aMCI.
Nouchi et al.^17^	114	One hundred‐forty‐four older adults participated in a 12‐week double‐blinded randomized control trial in which they were randomly assigned to one of four groups: the active control game (AT) with Sulforaphane (SFN) (AT‐S), the active control game with placebo (AT‐P), or the Brian training (BT) with Sulforaphane (SFN) (BT‐S) (AT‐P). Tetris in AT and Brain Age in BT. For 12 weeks, participants were required to play BT or AT for 15 min each day while also taking a supplement (SFN or placebo).	Contrary to the placebo intake groups, the SFN intake (BT‐S and AT‐S) groups shown significant gains in working memory and processing speed (BT‐P and AT‐P). But the researchers could discover no proof that the combined interventions had any positive benefits on cognition.
Wais et al.^18^	48	A total of 12 h of computer game play were randomly allocated to either the Labyrinth‐VR or placebo control game arms for 48 older persons (mean age 68.7, 6.4 years) with average cognitive capacities for their age. High‐fidelity LTM outcome measures were evaluated immediately before and after each participant's treatment regimen to evaluate mnemonic discrimination and other memory measures.	The Labyrinth‐VR arm's posttreatment high‐fidelity LTM competence increased in comparison to placebo, reaching levels comparable to those acquired by younger individuals in a different trial.
Bonnechère et al.^19^	12,000	This study assessed game scores and the processing speed achieved over the course of 100 sessions in 12,000 individuals aged 60 to over 80 years. Utilizing CMG, the cognitive performance was assessed.	Throughout the 100 sessions, users who trained with the games performed better independent of age in terms of scoring and processing speed, indicating that elderly and extremely old persons can enhance their cognitive ability with CMG.
Zhang et al.^20^	Initial intervention study = 25, Replication intervention study = 69	All participants were pretested in the laboratory over 3 days in 3 1‐h sessions, and then instructed to play their assigned video games at home for 45 h over a period of around 10 weeks. Finally, participants underwent a second posttest in the lab over the course of seven days in seven sessions of 1.5 h each. The N‐back task and the Attentional control task were used to assess learning performance.	This study emphasizes how action video game treatments may significantly boost cognitive task performance through faster learning for novel activities.
Anguera et al.^21^	31	The purpose of this study was to determine whether the improved cognitive abilities and probable brain alterations obtained via the usage of a specially created video game (NeuroRacer) played by older individuals (60–85 years old) remained higher beyond control participants 6 years later.	Beyond control participants, the NeuroRacer group continued to exhibit lower multitasking costs, and midline frontal theta power, a neural marker of cognitive control, continued to exhibit increased activity.
Kamnardsiri et al.^22^	5	An exercise prototype based on a game was developed in the development phase (Part I) by combining expertise, conducting literature research, and consulting experts on fall prevention exercise for older individuals. The result was a game‐based training prototype that addresses important physical and mental aspects of falls. Five games with three varying degrees of difficulty (Fruits Hunter, Where Am I?, Whack a Mole, Sky Falls, and Crossing Poison River) were evaluated in the usability testing (Part II) on five older individuals (mean age 70.40 years, SD 5.41 years). Participants evaluated their degree of satisfaction while playing the games using the Physical Activity Enjoyment Scale (PACES) and left comments about the games after finishing them. There were descriptive statistics.	According to the findings, the average PACES score was 123 out of 126 overall, and each item received a score between 6.66 and 7.00, suggesting a high degree of enjoyment. Positive feedback, such as admiration for the well‐designed interactions and user‐friendly interfaces, was also supplied.
Zhang et al.^23^	Yong = 20, Old = 16	For FTOMBVG, an electronic fingercot was created. Two sets of participants had their prefrontal cortex (PFC), motor cortex (MC), and occipital lobe (OL) functional near‐infrared spectroscopy (fNIRS) signals measuring oxygenated hemoglobin content (Delta [HbO]) recorded (old and young).	The young group only had unilateral activation in the cognitive area, whereas the older group displayed bilateral activation. The motor area was highly bilaterally activated in both groups. The old group's FC between the motor and cognitive regions was greatly improved.
Wang et al. ^24^	759	Randomized controlled trials were thoroughly searched for the meta‐analysis from 12 databases, trial registries, and gray literature sources. Utilizing Comprehensive Meta‐analysis Software 3.0, meta‐analysis and random‐effects meta‐regression were carried out. Hedges' *g* was used to calculate the overall effect, which was then calculated using Z‐statistics. The I2 and Cochran's Q tests were employed to look at heterogeneity. The overall quality of the evidence was assessed using the Grading of Recommendation, Assessment, Development, and Evaluation approach.	There were 759 older individuals included in 15 studies. According to a meta‐analysis, brain training using games significantly increased short‐term memory (*g* = 0.35), selective attention (*g* = 0.40), and processing speed (*g* = 0.23) when compared to a control group. Our subgroup analyses highlighted that a design with nontime‐pressure games, multiplayer, a computer platform, provider assistance, and sessions lasting no longer than 60 min each three times a week was the most effective. Game design was discovered by meta‐regression as having statistically significant effects on processing speed (*β* = 0.211, *p* = 0.008). Egger's regression asymmetry test revealed no publication bias (*p* = 0.293).

## 4 DISCUSSION

This literature review showed that positive effects were found in the elderly who underwent the computer/mobile phone‐based brain training game program. Some studies reported benefits not just in the psychological dimension, but also in the physical dimension.^22^ The variation in the studies depended on the type of brain training game provided, the measurement tools used, and the baseline brain competency (the level of decline in brain memory function) of the subjects. The setting that had more facility, expertise healthcare workers can be use more sensitive tools such as neurological test equipment. However, all studies reported positive effects on brain function, whatever, the different tools of measurements. Further studies about standard tools of brain function evaluation are needed. The limitation of this analysis is that the primary end point and the tools from each study were different, therefore, it is difficult to pool the result. Therefore, the meta‐analysis could not be applied in this analysis. A larger sample size and standardized measurement tools are needed to obtain more precise results and conclusions. The study designs also influenced the interpretation of the results. Given that many factors affect brain function, randomized controlled trials should be designed. However, the ideal clinical trial is difficult to implement because of its high expenditure. The use of statistics to decrease bias and control confounding factors may provide some advantages. Another point to consider is the limitation of generalized computer/mobile phone‐based brain training programs given that the technology knowledge of the elderly varies by area. Therefore, the computer/mobile phone‐based brain training game program should be provided in areas with suitable technology only. The competency of the community should be assessed before the computer/mobile phone‐based brain training game program. Some expert recommendations^5^, ^23^ regarding the potential of using this technology should also be followed given the presence of barriers in the elderly, such as reduced vision and loss of movement function speed. Such barriers are factors for consideration in the design of brain stimulation games.

## 5 CONCLUSION

The brain training game showed a positive effect on the brain function. However, the confirmation studies with large populations and standard measurement tools should be developed for more validated results.

## AUTHOR CONTRIBUTIONS

**Karunrat Tewthanom**: Conceptualization; data curation; formal analysis; investigation; writing—original draft; writing—review and editing. **Sittha Sukasi**: Conceptualization; investigation; writing—review and editing. **Sarawut Lerspalungsanti**: Conceptualization; investigation; writing—review and editing. **Foifon Srisawat**: Conceptualization; investigation; writing—review and editing.

## CONFLICT OF INTEREST STATEMENT

The authors declare no conflict of interest.

## TRANSPARENCY STATEMENT

The lead author Sittha Sukasi affirms that this manuscript is an honest, accurate, and transparent account of the study being reported; that no important aspects of the study have been omitted; and that any discrepancies from the study as planned (and, if relevant, registered) have been explained.

## Data Availability

The authors confirm that the data supporting the findings of this study are available within the article.
